# Bacterial phytopathogen infection disrupts belowground plant indirect defense mediated by tritrophic cascade

**DOI:** 10.1002/ece3.3052

**Published:** 2017-05-26

**Authors:** Monique J. Rivera, Kirsten S. Pelz‐Stelinski, Xavier Martini, Lukasz L. Stelinski

**Affiliations:** ^1^ Entomology and Nematology DepartmentCitrus Research and Education CenterUniversity of FloridaLake AlfredFLUSA; ^2^ Entomology and Nematology DepartmentNorth Florida Research and Education CenterUniversity of FloridaQuincyFLUSA

**Keywords:** belowground tritrophic interactions, entomopathogenic nematodes, herbivore‐induced plant volatiles, huanglongbing, plant–insect interactions, soil ecology

## Abstract

Plants can defend themselves against herbivores through activation of defensive pathways and attraction of third‐trophic‐level predators and parasites. Trophic cascades that mediate interactions in the phytobiome are part of a larger dynamic including the pathogens of the plant itself, which are known to greatly influence plant defenses. As such, we investigated the impact of a phloem‐limited bacterial pathogen, *Candidatus* Liberibacter asiaticus (*C*Las), in cultivated citrus rootstock on a well‐studied belowground tritrophic interaction involving the attraction of an entomopathogenic nematode (EPN), *Steinernema diaprepesi*, to their root‐feeding insect hosts, *Diaprepes abbreviatus* larvae. Using belowground olfactometers, we show how *C*Las infection interferes with this belowground interaction by similarly inducing the release of a C12 terpene, pregeijerene, and disconnecting the association of the terpene with insect presence. *D. abbreviatus* larvae that were not feeding but in the presence of a *C*Las‐infected plant were more likely to be infected by EPN than those near uninfected plants. Furthermore, nonfeeding larvae associated with *C*Las‐infected plants were just as likely to be infected by EPN as those near noninfected plants with *D. abbreviatus* larval damage. Larvae of two weevil species, *D. abbreviatus* and *Pachnaeus litus*, were also more attracted to plants with infection than to uninfected plants. *D. abbreviatus* larvae were most active when exposed to pregeijerene at a concentration of 0.1 μg/μl. We attribute this attraction to *C*Las‐infected plants to the same signal previously thought to be a herbivore‐induced plant volatile specifically induced by root‐feeding insects, pregeijerene, by assessing volatiles collected from the roots of infected plants and uninfected plants with and without feeding *D. abbreviatus*. *Synthesis*. Phytopathogens can influence the structuring of soil communities extending to the third trophic level. Field populations of EPN may be less effective at host‐finding using pregeijerene as a cue in citrus grove agroecosystems with high presence of *C*Las infection.

## 1 INTRODUCTION

Plants, as immobile organisms, are often exposed to multiple stressors. Stressors can be herbivores, pathogens, parasites and abiotic conditions or some combination of those occurring either simultaneously or sequentially both above‐ and belowground. Plants cannot actively avoid stressors but can feature innate or induced defensive phenotypes. Enhanced pest tolerance over the lifetime of a plant can depend on physical structures to prevent attacks and internally the production of defensive secondary compounds in response to stressor exposure. Plant secondary compounds are downstream products of molecular pathway activation and can mediate plant interactions with their biotic and abiotic environments (Ozawa, Arimura, Takabayashi, Shimoda, & Nishioka, 2000; Pieterse & Dicke, 2007). With specific regard to insect herbivores which feed on the plant, many of these compounds are toxic and repellant, but when released as volatiles can also be utilized by herbivore predators and parasites to locate hosts (Mumm & Dicke, 2010b). Based on the metabolic cost implied in the production of secondary metabolites, many studies have investigated variation in production by classifying their modes of production as “constitutive” or “inducible” despite evidence that these defenses are not mutually exclusive (e.g., Zangerl & Berenbaum, 1990). There has been a larger focus on induced chemistry likely because it represents a clear response to attack.

Throughout the plant, induced responses can be produced locally at the feeding site or systemically (Bezemer & Van Dam, 2005; Kaplan, Halitschke, Kessler, Sardanelli, & Denno, 2008). The production of induced defenses is activated and regulated by phytohormone pathways such as jasmonic acid, salicylic acid, and ethylene (Pieterse & Dicke, 2007). Phytohormones regulate a suite of induced plant responses, which include the production herbivore‐induced plant volatiles (HIPVs; Walling, 2000). Pathways that produce HIPVs are highly conserved among plants and are often activated differentially depending on the type of damage incurred by plants (Zipfel, 2014). For example, insect feeding guild (piercing and sucking versus chewing) influences which pathways are activated (Walling, 2000; Wu & Baldwin, 2009; Zarate, Kempema, & Walling, 2007).

Plants also affect the phytobiome by changing the surrounding volatile profile both above‐ and belowground (Dicke, 2016). The ability of plants to facilitate attraction of herbivore predators and parasites with HIPVs has been shown in multiple plant species. Predators and parasites can exhibit top‐down control of insect herbivore populations above‐ and belowground which can regulate the overall impact of herbivory on plants (van Dam, Qiu, Hordijk, Vet, & Jansen, 2010; Dicke & Baldwin, 2010; Kessler & Baldwin, 2000; Preisser, Dugaw, Dennis, & Strong, 2006). Changes in volatile profile due to insect feeding can influence plant‐beneficial tritrophic interactions by attracting tertiary predators and parasites of insect herbivores which is known as indirect defense (Mumm & Dicke, 2010a; Price et al., 1980). This has been widely investigated in aboveground environments (e.g., De Moraes, Mescher, & Tumlinson, 2001; Meiners & Hilker, 1997) and more recently in belowground environments (Ali, Alborn, & Stelinski, 2010; Rasmann et al., 2005).

There has been growing interest in exploiting such multitrophic interactions for biological pest control in agriculture (Bommarco, Kleijn, & Potts, 2013). For belowground pest species, the interest has been in exploiting the attraction of entomopathogenic nematodes (EPN) to damaged plants for location and infection of root‐feeding herbivores. This tritrophic interaction has been shown in multiple systems (Aratchige, Lesna, & Sabelis, 2004; van Tol et al., 2001). Studies using maize and citrus have demonstrated this interaction both in the laboratory (Ali et al., 2010; Rasmann et al., 2005) and directly in agroecosystems (Ali et al., 2012; Degenhardt et al., 2009). However, a central unknown of manipulating a natural environment is how the interactive effects of multiple plant stressors impact belowground tritrophic interactions. Agricultural systems, while disturbed, are useful model systems for investigating the effects of multiple stressors on plants and their related interactions because of the redundancy of pests and pathogens and, thus, the predictability of the species occurring in these environments. We used the citrus huanglongbing (HLB) pathosystem to investigate the effects of a phloem‐limited bacterial infection on a belowground tritrophic interaction mediated by a HIPV, pregeijerene.

Huanglongbing is caused by the gram‐negative bacterium, *Candidatus* Liberibacter asiaticus (*C*Las), which is transmitted by the Asian citrus psyllid (ACP; *Diaphorina citri*). HLB is the most damaging disease of cultivated citrus worldwide (da Graca et al., 2016). The vector is attracted to infected trees following induced changes in tree volatile production, which may promote spread of disease (Mann et al., 2012; Mauck, Moraes, & Mescher, 2016). *C*Las is quickly moved throughout the plant via phloem translocation; the roots have the third highest accumulation of the bacteria of all plant tissues (Tatineni et al., 2008). This suggests that activation of defense pathways may occur throughout the plant, including in the roots, which may influence HIPVs released from the plant upon secondary attack.

In the citrus rhizosphere, the C12 terpene compound, pregeijerene, attracts soil‐dwelling infective juveniles (IJs) of multiple EPN species to feeding *D. abbreviatus* larvae. These larvae chew on the plant's roots, which stimulates an induced release of pregeijerene into the soil (Ali et al., 2012). EPNs are attuned to HIPVs as cues in their specific plant system for host‐finding through exposure over time (Hiltpold, Baroni, Toepfer, Kuhlmann, & Turlings, 2010; Rasmann, Ali, Helder, & van der Putten, 2012; Willett, Alborn, Duncan, & Stelinski, 2015). We hypothesized that *C*Las infection may impact belowground release of HIPVs and investigated how this may impact EPN response to feeding *D. abbreviates* larvae in the citrus root zone.

## 2 METHODS

### 2.1 Insects, nematodes, and plants

#### 2.1.1 Insects

Noninfected adult *D. citri* used to feed on plants as a damage treatment were obtained from a laboratory culture at the University of Florida, Citrus Research and Education Center (Lake Alfred, USA). The culture was established from field populations in Polk Co., Florida, USA (28.09N, 81.99W), before the local discovery of HLB in Florida. *D. citri* colonies were kept in double‐screened, 3.764‐m secure enclosures in an air‐conditioned glasshouse at 27–28°C, 60%–65% RH, and L14:D10 photoperiod. Bimonthly testing of randomly sampled adults and plants by quantitative PCR (qPCR) assays was conducted to confirm that psyllids and plants in this culture were uninfected by *C*Las.

*Diaprepes abbreviatus* and *Pachnaeus litus* larvae were obtained from a laboratory colony maintained at University of Florida's Citrus Research and Education Center (CREC) in Lake Alfred, FL. This culture was periodically supplemented from a larger colony maintained at the Division of Plant Industry Sterile Fly Facility in Gainesville, FL. Larvae were reared on a commercially prepared diet (Bio‐Serv, Inc., Frenchtown, NJ, USA) using procedures described by Lapointe and Shapiro (1999). Larvae used in experiments were from third to sixth instars.

#### 2.1.2 Nematodes

The entomopathogenic nematode, *S. diaprepesi* HK3, was initially isolated from *D. abbreviatus* larvae in Florida commercial citrus orchards. *S. diaprepesi* was reared in larvae of the greater wax moth, *Galleria mellonella*, at approximately 25°C according to procedures described in Kaya and Stock (1997). Infective juveniles emerged from insect cadavers into emergence traps and were then transferred to and stored in shallow water in tissue culture flasks at 15°C in an incubator for up to 2 weeks before use in experiments.

#### 2.1.3 Plants

“Swingle citrumelo” (*Citrus paradisi* Macf.* *× *Poncirus trifoliata* L. Raf.) rootstock is among the most common commercially grown citrus varieties. Given its relevance to production and use in previous studies involving belowground tritrophic interactions, this variety was selected for use in experiments. All plants were grown and maintained at CREC in Lake Alfred, FL, in a glasshouse at 26°C and 60%–80% RH.

Las infection in host plants was established by graft inoculation of noninfected Swingle plants with *C*Las‐infected Swingle plant material collected from citrus groves in Immokalee, FL, USA. Grafted plants were tested for Las infection using qPCR 4 months after grafting to allow for ample time for the transfer of infection. *C*Las‐positive plants were used in experiments and were allowed 6 months to return to their predamage state before use in experiments (Metlen, Aschehoug, & Callaway, 2009). Graft inoculations, as opposed to inoculation by *D. citri*, were used to control the quantity of pathogen transmitted to each plant. *C*Las‐free host plants used in experiments were grown from *C. sinensis* seed or obtained as potted seedlings from an HLB‐free commercial nursery to minimize the risk of undetectable latent infection of *C*Las in grafted plants. The nursery‐obtained plants were confirmed negative for *C*Las infection by qPCR. All infected plants used for experiments exhibited minor or no symptoms, ranging from 0 to 1 on a graded symptom scale of 1–10. Noninfected and *C*Las‐infected plants were maintained in separate secure enclosures with minimal risk of cross contamination as described above. All plants were infected 4 months before use in experiments.

Plants were <1 year old at the time of experiments. When plants are referred to as mechanically damaged, this was done to the plant by puncturing the leaf tissues with a Minuten insect pin approximately 20 times. Plants referred to as damaged were exposed to larval feeding for 48 hr prior to assays.

### 2.2 Effect of *C*Las infection on subterranean host‐seeking behavior of herbivores and their parasites

In order to unravel the interactive effect of *C*Las infection in plants on belowground first‐ and second‐level trophic interactions, a series of experiments was performed using glass, soil‐filled olfactometers described in detail by Ali, Alborn, and Stelinski (2011). In brief, six‐arm olfactometers (Ali et al., 2011) were modified into four‐choice chambers by closing off two of the six arms. Glass olfactometers were further modified by removing all mesh screens to allow entomopathogenic nematodes access to all parts of the olfactometer. Treatment plants were placed in the distal portion of all arms of the olfactometer along with a single fifth‐instar *D. abbreviatus* larva in certain treatments. Aliquots consisting of either 30* *μl pentane (blank control) or various solutions of pregeijerene (described below) were placed on filter paper and added to the root zone in each of the two arms. The olfactometer was then filled with washed autoclaved silica sand adjusted to 10% moisture by volume.

#### 2.2.1 Weevils: *Diaprepes abbreviatus* and *Pachnaeus litus*

Root herbivores, *D. abbreviatus* and *P. litus*, were used to assess changes in belowground herbivore assays in response to plants with *C*Las infection and/or herbivore feeding damage. A single weevil larva of either species was released and allowed 24 hr to choose between the two treatments. At the end of 24 hr, choice was designated by the presence of the larva in the chamber at either end of the olfactometer. To exclude directional bias or possible inherent preference between plants, the initial olfactometer treatment compared beetle response between two undamaged and uninfected plants for both species.

*Diaprepes abbreviatus* larvae were given the choice between two plants with no infection or two plants with *C*Las infection. These two‐choice tests were also randomly subjected to the treatments given below. There were two experiments using plants with no *C*Las infection where the larvae were provided the following choices: no damage versus mechanical damage and no damage versus *D. abbreviatus* damage. Likewise, there were two experiments using plants with *C*Las infection that compared the following treatments: no damage versus ACP feeding and no damage versus *D. abbreviatus* damage. Each of these individual comparisons was replicated 30 times.

To determine whether the effects are relevant broadly across weevil species, *P. litus* larvae were also investigated. Larvae were also placed individually into a two‐choice olfactometer, and three experiments were conducted with undamaged plants: no infection versus no infection, no infection versus *C*Las infection, and one with no plants comparing response to 0.1 μg/μl pregeijerene versus a solvent blank of dichloromethane. Each of these individual comparisons was replicated 30 times.

#### 2.2.2 Entomopathogenic nematode: *Steinernema diaprepesi*

To assess the interactive effect of *C*Las infection and insect damage on the attraction of EPN to their insect hosts, 2,500 *S. diaprepesi* infective juveniles (IJs) were released into a two‐choice setting containing various sets of two treatments. Nematodes were allowed to forage for 24 hr before the olfactometer was disassembled. Nematodes were extracted from the substrate over 48 hr using Baermann funnels and counted using a dissection microscope. The treatments investigated mirrored those tested with *D. abbreviatus* larvae. The treatments overlaid in experiments with two plants without *C*Las infection were no damage versus mechanical damage and no damage versus *D. abbreviatus* damage. Using plants with *C*Las infection, we tested the following: no damage versus *D. citri* feeding and no damage versus *D. abbreviatus* damage. Likewise, IJs were assayed with two plants without infection, as well as two plants with *C*Las infection that were otherwise unmanipulated. Each experiment was replicated 30 times.

#### 2.2.3 Infestation and infection

Six‐arm olfactometers were modified to create a four‐choice design by closing off access to two of the six arms, as described in Ali et al. (2010), and only attaching four outer chambers containing treatments, as described above. Treatments were placed randomly among the four arms. *D. abbreviatus* larvae were placed into stainless steel screen cages (1‐cm^2^ cubes, 225 mesh) that prevented feeding on roots but allowed entry of nematodes; there was one caged larva per arm. At the onset of the experiment, 2,500 *S. diaprepesi* IJs were released into the central chamber and allowed to forage for 48 hr. Two experimental designs were replicated 20 times each. In both experiments, the caged larva in each treatment arm was combined with a treatment applied to the plant or a blank. The first experiment consisted of four treatments paired with a caged larva: an uninfected plant, a plant with *C*Las infection, and two blanks with nothing added to these treatment arms except for the caged larvae. In the second experiment, nematode response was tested to a *C*Las‐infected plant as compared with an uninfected plant that was actively damaged by *D. abbreviatus* larval feeding. Two additional blank (negative control) treatment arms completed the four possible directions nematodes could forage. Nematodes were collected and counted as described above. Also, caged larvae were collected to determine infestation by EPNs as measured by subsequently emerging IJs and larval mortality as described in Ali et al. (2012).

### 2.3 Dose–response

A dose–response curve was developed to determine the most active dosage of pregeijerene to attract *D. abbreviatus* larvae under our olfactometer experiential conditions. Pregeijerene was obtained as described in Ali et al. (2011). In the olfactometer, the larvae were given a choice between a dichloromethane blank and one of five logarithmically doses of pregeijerene dissolved in this solvent. Volatiles were released from filter paper as described in Ali et al. (2011). Larvae were scored as making a choice when they moved into either of the chambers containing treatments in the olfactometer 48 hr after release. Larvae were collected by disassembling olfactometers and sifting removed sand in the radiating treatment arms.

### 2.4 Induced release of pregeijerene

Volatiles were collected from the following plants to assess the presence or absence of pregeijerene: an HLB‐infected plant, an uninfected plant damaged by *D. abbreviates* larval root feeding as described above, a plant with both *C*Las infection and *D. abbreviatus* damage, and for comparison, an uninfected and uninfected plant, and volatile samples of the substrate itself. In each case, the presence of pregeijerene was verified with an authentic standard (CAS 020082‐17‐1). Each treatment was replicated five times.

Baseline volatile production was determined by initially sampling the volatiles from the roots of both plants three days before treatment was applied for comparison. On day 4, plants were infested with one larva at the root zone. Noninfested plants were not exposed to weevils during this period.

Plants were potted in sand‐filled glass root‐zone chambers as previously described in Ali et al. (2011). Seedlings were given 3 days to adjust to their sand‐filled chambers. Infested plants were subjected to an additional 3 days of feeding by weevil larvae. The belowground chambers of each infestation type were simultaneously sampled for three subsequent days after infestation. Root feeding damage by beetle larvae was confirmed visually as described in Ali et al. (2011).

Each root‐zone chamber was connected to a vacuum pump (ARS) for 24 hr with a suction flow of 80 ml/min (Ali et al., 2011). Compounds emitted from chambers were collected on adsorbent traps filled with 50 mg Super‐Q (800–1,000 mesh; Alltech Assoc.) held in glass fittings between the chamber and vacuum pump (Ali et al., 2011). Thereafter, Super‐Q traps were rinsed with 150 L of dichloromethane into individual 2.0‐ml clear glass vials (Varian, Palo Alto, CA, USA, part number: 392611549, equipped with 500‐L glass inserts) (Ali et al., 2011).

All samples were injected as 1‐μl aliquots of dichloromethane extracts onto a Clarus 500 GC/MS (PerkinElmer, Shelton, CT, USA) equipped with a DB1 capillary column with 30 m length, 0.25 mm internal diameter, and 0.25 mm film thickness (Quadrex, New Haven, CT, USA). Root‐zone collection chambers used to collect belowground volatiles were filled with heat‐sterilized sand standardized at 10% saturation. Samples were introduced by splitless injection at 220°C. The temperature was as follows: The column was held at 50°C for 3 min and then increased to 200°C at a rate of 4°C/min and held for 5 min. Helium was the carrier gas with flow rate 1 ml/min. Pregeijerene was identified by comparing its retention time, linear retention indices, and the selected ions with those of an authentic standard. Although α‐santalene and α*‐Z*‐bergamotene were present at lower detection levels in *Diaprepes‐*damaged and *C*Las‐infested treatments as reported previously by Ali et al. (2010), we focused on the presence/absence of pregeijerene as this was the dominant HIPV and has been decisively proven as the behaviorally active HIPV for the nematode species investigated (Ali et al., 2012).

### 2.5 Statistical analyses

In olfactometer experiments where two‐choice responses of beetle larvae were investigated, McNemar's tests were used to analyze data. Data from two‐choice experiments with entomopathogenic nematode response as the dependent variable were converted to percent response. These data were then analyzed using paired *t* tests after data were analyzed for normality. Both McNemar's test and the *t* tests were performed using GraphPad Prism 7 (GraphPad Software Inc., San Diego, CA, USA). The four‐choice olfactometer experiments with nematode infection as the dependent variable were analyzed using Proc Genmod (SAS 9.3, Cary, NC) with a binomial distribution. *D. abbreviatus* response to logarithmic increases in dose of pregeijerene was analyzed using two generalized linear models with a binomial distribution. The first assessed whether the proportion of *D. abbreviatus* larvae changed in response to the dose treatment, and the second compared the number of responding larvae by treatment. These analyses were performed using R (version 3.1.3; The R Foundation for Statistical Software, Vienna, Austria).

## 3 RESULTS

### 3.1 Effect of interaction between infection and damage: *Diaprepes abbreviatus*

**Figure 1:**
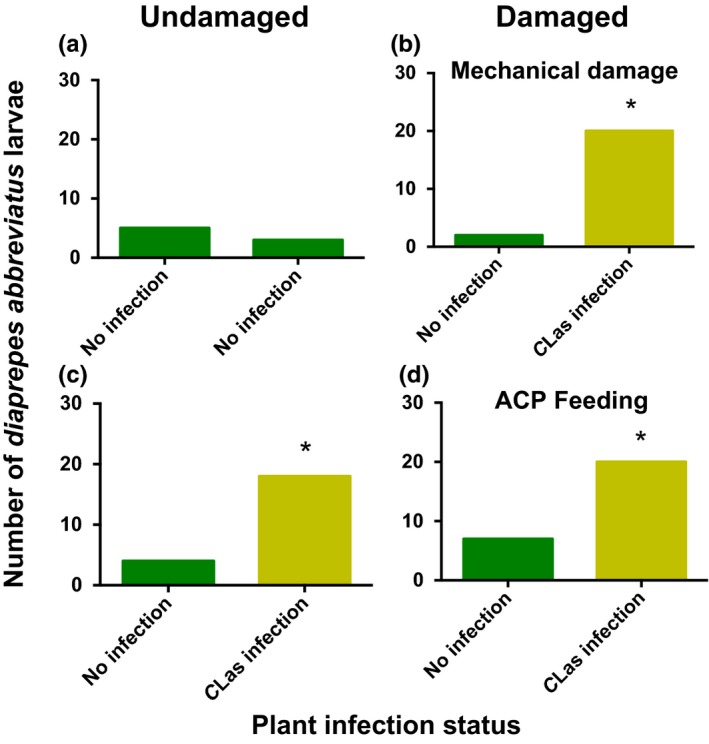
Effect of herbivore or mechanical damage to *Candidatus* Liberibacter asiaticus (*C*Las)‐infected or uninfected citrus plants [“Swingle citrumelo” (*C. paradisi* × *P. trifoliata*) rootstock] on behavior of larval *Diaprepes abbreviatus*. A single *D. abbreviatus* larva was released in a two‐choice olfactometer to assess attraction to treatments (*n* = 30). Comparisons were conducted between two (a) undamaged and uninfected plants, (b) mechanically damaged plants with or without *C*Las infection, (c) *C*Las‐infected plants, and (d) plants with *Diaphorina citri* (ACP) feeding with or without *C*Las infection. Total number of *D. abbreviatus* larvae attracted without an asterisk did not differ significantly (α = .05)

*Diaprepes abbreviatus* larvae responded equally between two undamaged plants without *C*Las infection confirming an unbiased response in the olfactometer (χ^2^ = 0.125; *df* = 1; *p* = .7237; Figure 1a). More *D. abbreviatus* larvae were attracted to *C*Las‐infected plants over uninfected plants with additional mechanical damage (χ^2^ = 10.050; *df* = 1; *p* = .0001; Figure 1b) or ACP feeding (χ^2^ = 5.333; *df* = 1; *p* = .0209; Figure 1d). Also, more larvae were attracted to *C*Las‐infected plants than to uninfected plants without additional mechanical damage (χ^2^ = 7.268; *df* = 1; *p* = .0056; Figure 1c).

**Figure 2:**
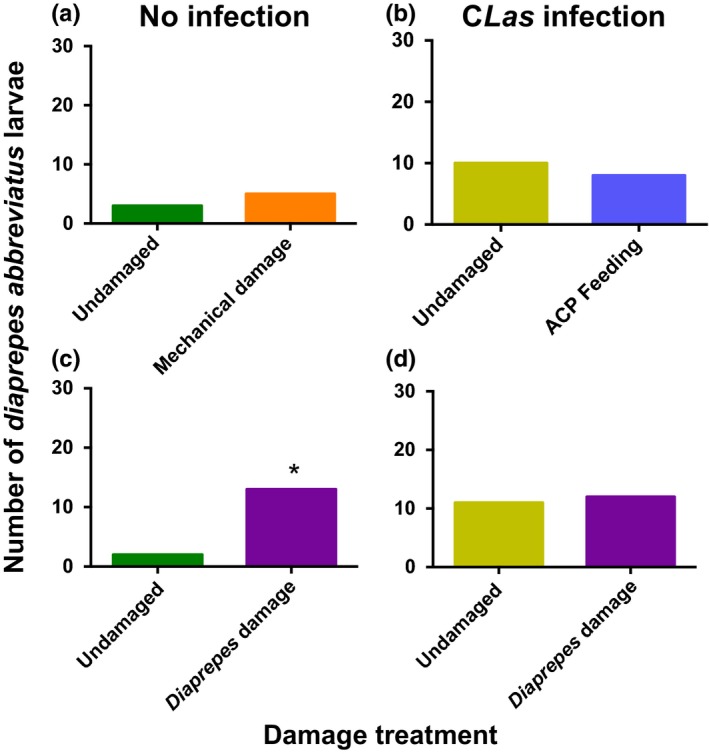
Effect of *Candidatus* Liberibacter asiaticus (*C*Las) infection on behavior of larval *Diaprepes abbreviatus* in response to mechanical damage, *Diaphorina citri* (ACP) feeding, and larval *Diaprepes abbreviatus* damage to citrus plants [“Swingle citrumelo” (*C. paradisi* × *P. trifoliata*) rootstock]. A single *D. abbreviatus* larva was released in a two‐choice olfactometer to assess attraction to treatments (*n* = 30). Attraction to (a) uninfected plants with or without mechanical root damage, (b) *C*Las‐infected plants with or without *Diaphorina citri* (ACP) feeding damage, (c) uninfected plants with or without *D. abbreviatus* feeding root damage, and (d) *C*Las‐infected plants with or without *D. abbreviatus* feeding root damage. Total numbers of *D. abbreviatus* larvae attracted without an asterisk did not differ significantly (α = .05)

Larval beetles exhibited no preference between uninfected plants with mechanical damage and undamaged plants that were also not infected with *C*Las (χ^2^ = 0.125; *df* = 1; *p* = .7237; Figure 2a). There was also no preference between *D. citri*‐damaged and undamaged plants that were also *C*Las‐infected (χ^2^ = 0.056; *df* = 1; *p* = .8137; Figure 2b). However, more *D. abbreviatus* were attracted to *D. citri*‐damaged plants than to undamaged plants when they were otherwise uninfected by the pathogen (χ^2^ = 6.667; *df* = 1; *p* = .0098; Figure 2c). *D. abbreviatus* did not exhibit preference for plants with *D. abbreviatus* larval damage as compared with undamaged plants when both plants were also infected with *C*Las (χ^2^ = 0.000; *df* = 1; *p* = 1.000; Figure 2d).

### 3.2 Effect of interaction between infection and damage: *Pachnaeus litus*

**Figure 3:**
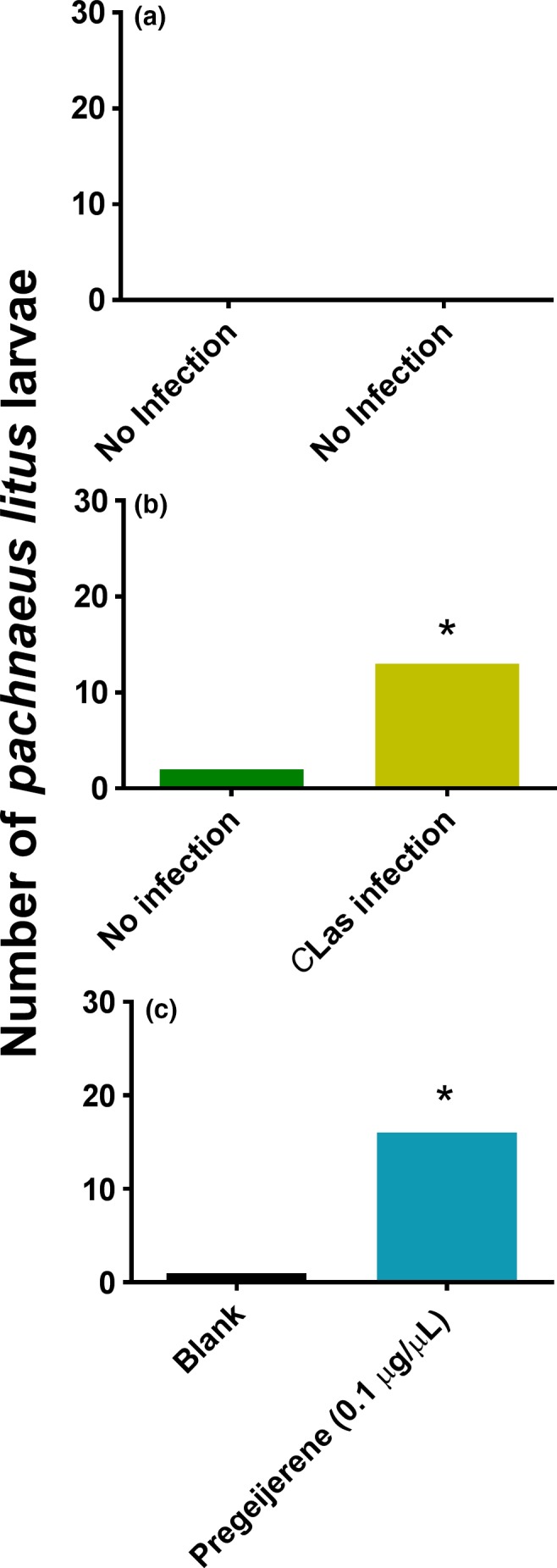
Attraction of *Pachnaeus litus* larvae to *Candidatus* Liberibacter asiaticus (*C*Las)‐infected plants or pregeijerene. A single *P. litus* larva was released in a two‐choice olfactometer to assess attraction to treatments (*N* = 30). Attraction to (a) two uninfected plants, (b) an uninfected plant versus an infected plant, and (c) pregeijerene (0.1 μg/μl) versus a solvent blank. Total numbers of *P. litus* larvae attracted without an asterisk did not differ significantly (α = .05)

*P. litus* responded equally between two undamaged plants without *C*Las infection (Figure 3a). *P. litus* exhibited preference for *C*Las‐infected plants as compared with uninfected plants that were otherwise undamaged (χ^2^ = 6.667; *df* = 1; *p* = .0098; Figure 3b). In the presence of no plants, *P. litus* was attracted to pregeijerene (0.1 μg/μl) as compared with a solvent blank (χ^2^ = 11.529; *df* = 1; *p* = .0007; Figure 3c).

### 3.3 Effect of interaction between infection and damage: *Steinernema diaprepesi*

**Figure 4:**
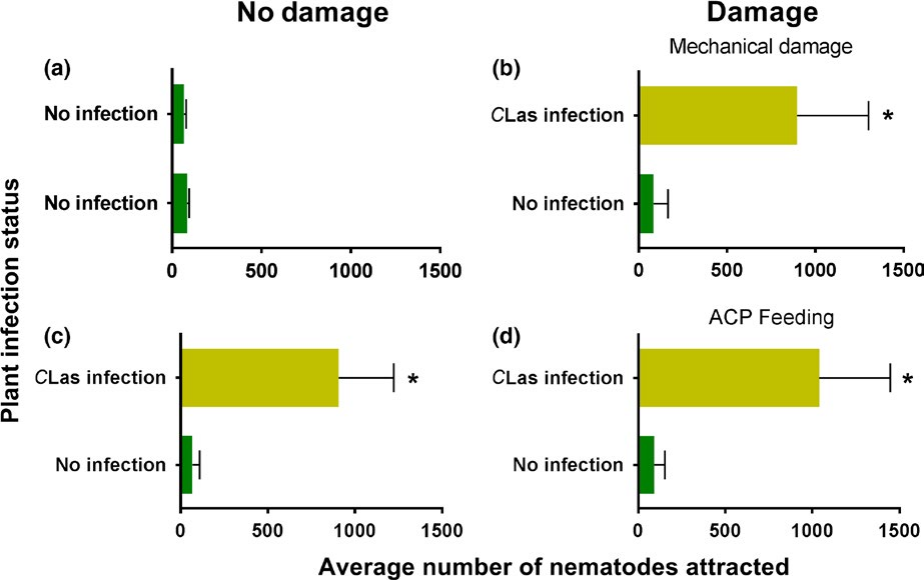
Effect of damage on entomopathogenic nematode (*Steinernema diaprepesi*) attraction to *Candidatus* Liberibacter asiaticus (*C*Las) infection in citrus plants [“Swingle citrumelo” (*C. paradisi* × *P. trifoliata*) rootstock]. Attraction was assessed using a two‐choice olfactometer and allowing 2,500 *S. diaprepesi* infective juveniles to forage for 24 hr before counting the number of nematodes associated with each of the treatments (*n* = 20). Comparisons were conducted between two (a) uninfected and undamaged plants, (b) plants mechanically damaged with Minuten pins to simulate aboveground feeding by *Diaphorina citri* (ACP) with or without *C*Las infection, (c) undamaged plants with or without *C*Las infection, and (d) plants with *D. citri* (ACP) feeding damage on aboveground plant parts with or without *C*Las infection. Mean (+*SEM*) number of nematodes without an asterisk did not differ significantly (α = .05)

*S. diaprepesi* infective juveniles showed no preference when challenged with odors of two similar undamaged and uninfected plants confirming no positional bias in the olfactometer (*t* = 1.066; *df* = 19; *p* = .1499; Figure 4a). More nematodes were attracted to undamaged plants that were *C*Las‐infected than uninfected plants (*t* = 11.58; *df* = 19; *p* = .0001; Figure 4c). Mechanical damage (*t* = 8.566; *df* = 19; *p* = .0001; Figure 4b) and *D. citi* feeding (*t* = 10.55; *df* = 19; *p* = .0001; Figure 4d) did not affect the behavior of nematodes to infected as compared with uninfected plants.

**Figure 5:**
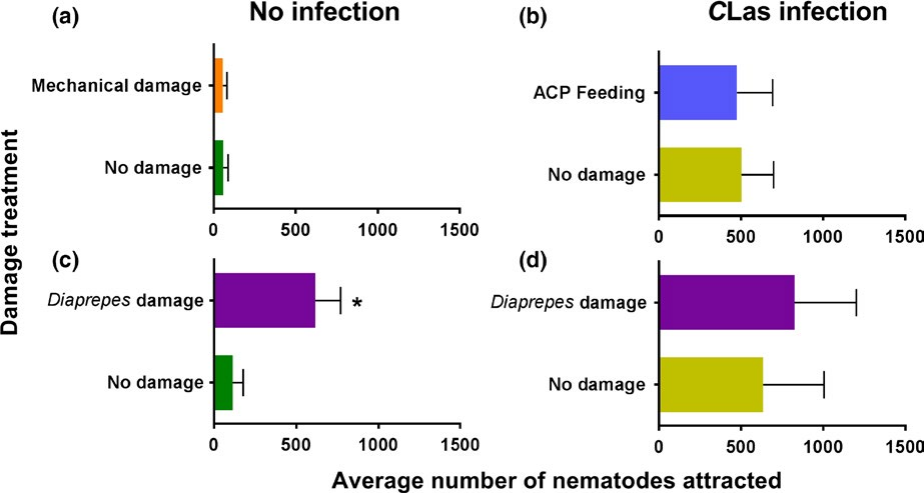
Effect of *Candidatus* Liberibacter asiaticus (*C*Las) infection on entomopathogenic nematode (*Steinernema diaprepesi*) attraction to three different types of damage to citrus plants [“Swingle citrumelo” (*C. paradisi* × *P. trifoliata*) rootstock]. Attraction was assessed using a two‐choice olfactometer and allowing 2,500 *S. diaprepesi* infective juveniles to forage for 24 hr before counting the number of nematodes associated with each of the treatments (*n* = 20). Attraction to (a) uninfected plants with or without mechanical damage, (b) *C*Las‐infected plants with or without *Diaphorina citri* (ACP) feeding damage, (c) uninfected plants with or without *Diaprepes abbreviatus* damage, and (d) *C*Las‐infected plants with or without *D. abbreviatus* feeding damage. Mean (+*SEM*) number of nematodes without an asterisk did not differ significantly (α = .05)

Nematodes exhibited no preference between mechanically damaged and undamaged plants in the absence of pathogen infection (*t* = 0.2121, *df* = 19, *p* = .4172; Figure 5a). However, uninfected plants with *D. abbreviatus* larval feeding damage on the roots were more attractive to nematodes than plants with no larval feeding damage (*t* = 12.06; *df* = 19; *p* = .0001; Figure 5c). When plants were infected with *C*Las*,* nematodes exhibited no preference between plants damaged by *D. citri* adults on aboveground plant tissues and undamaged plants (*t* = 0.4140; *df* = 19; *p* = .6835; Figure 5b). Also, nematodes exhibited no preference between *C*Las‐infected plants that were damaged by *D. abbreviatus* larval feeding on belowground roots and those receiving no damage (*t* = 1.273; *df* = 19; *p* = .1092; Figure 5d).

### 3.4 *D. abbreviatus* infestation and infection

**Figure 6:**
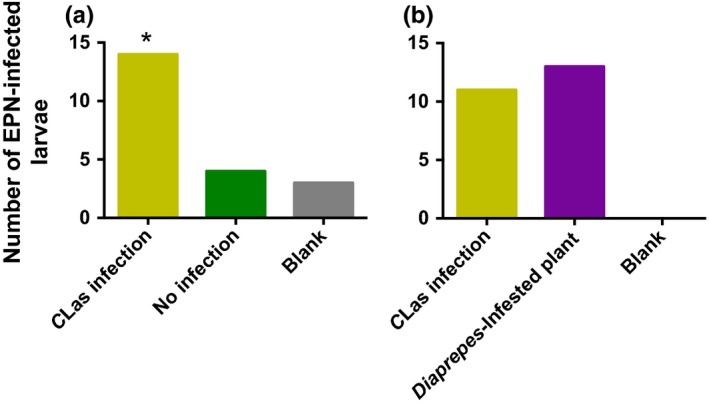
Effect of *Candidatus* Liberibacter asiaticus (*C*Las) infection in citrus plants [“Swingle citrumelo” (*C. paradisi* × *P. trifoliata*) rootstock] on host location by *Steinernema diaprepesi*. (EPN). No active feeding by the weevil larvae occurred during these experiments. *S. diaprepesi* (2,500 infective juveniles) were released into four‐choice belowground olfactometers and allowed to forage for 48 hr. Each treatment contained one caged *Diaprepes abbreviatus* larva as a sentinel for EPN infection. EPN response to (a) uninfested and uninfected plant, a plant with *C*Las infection, or two blank arms, and (b) *C*Las‐infected plant, plant with *D. abbreviatus* damage, or two blank treatment arms (*n* = 20). Total numbers of infected larvae per treatment without an asterisk did not differ significantly (α = .05)

More *D. abbreviatus* larvae were infected by EPNs when in the presence of *C*Las‐infected than uninfected plants (χ^2^ = 9.68; *df* = 3; *p* = .0079; Figure 6a). Nematode infection of *D. abbreviatus* larvae was similar in the presence of plants infected with *C*Las that received no additional feeding damage from *D. abbreviatus* and those that were uninfected by pathogen, but that were actively infested with larval *D. abbreviatus*; however, both of these treatments resulted in greater EPN infection of *D. abbreviatus* larvae than blank controls (χ^2^ = 15.16; *df* = 3; *p* = .0041; Figure 6b).

### 3.5 Optimal dosage of pregeijerene for attraction of *D. abbreviatus*

**Figure 7:**
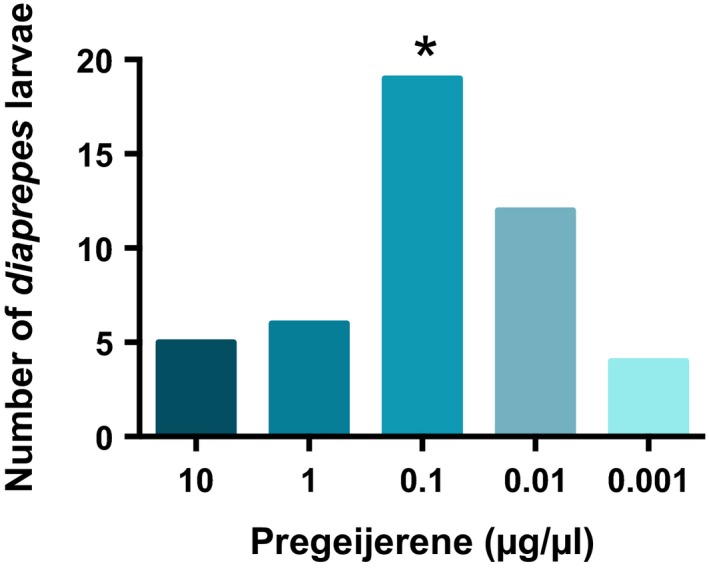
Response of *Diaprepes abbreviatus* larvae to five logarithmically increasing doses of pregeijerene. A two‐choice olfactometer was used to assess response of larvae. Experiments with each dose were replicated 30 times. The mean number of larvae moving toward the blank was 1.8. The optimal dose for *D. abbreviatus* attraction was 0.1 μg/μl (z = 3.011; *df* = 4; *p* = .0026). Total numbers of larvae per treatment without an asterisk did not differ significantly (α = .05)

Attraction of *D. abbreviatus* to pregeijerene did not increase proportionally in a stepwise pattern with log dosage (χ^2^ = 0.5880; *df* = 4; *p* = .4430). However, significantly more *D. abbreviatus* responded toward pregeijerene at the 0.1 μg/μl concentration as compared with the solvent control (z = 3.011; *df* = 4; *p* = .0026; Figure 7). To more specifically predict the optimal concentration for attraction, the coefficients were used to calculate 0.045 μg/μl (y = ‐1.22882 × *x* + 0.11219 × *x* ^2^‐0.12148).

### 3.6 Induced release of pregeijerene

As shown previously (Ali et al., 2010), pregeijerene release from plant roots damaged by *D. abbreviatus* larvae was confirmed (Table 1). Pregeijerene was also emitted from plants infected with *C*Las regardless of additional *D. abbreviatus* larval damage (Table 1). α‐Santalene and α*‐Z*‐bergamotene were the only two other volatiles detected in *Diaprepes*‐damaged and *C*Las‐infected plants (data not shown); however, these do not affect EPN behavior (Ali et al., 2010).

**Table 1:** Pregeijerene release from *C*Las‐infected or *Diaprepes abbreviates*‐damaged citrus plant roots

Treatment	Pregeijerene presence
*Candidatus* Liberibacter asiaticus (CLas)‐infected plant	+
Uninfected and *Diaprepes abbreviatus*‐infested plant	+
CLas‐infected and *D. abbreviates*‐infested plant	+
Uninfected and uninfested plant	−
Blank	−

## 4 DISCUSSION

We investigated how multiple stressors impact plant response and possibly influence the structure of plant‐associated communities. Previous investigations of belowground tritrophic interactions involving entomopathogenic nematodes (EPNs) used undamaged, disease‐free plants in various artificial environments where multiple stressors were least likely to be present. The intention of these investigations was to isolate the interaction of interest, which has been extremely important in unraveling these multitrophic interactions (Ali et al., 2012; Filgueiras, Willett, Junior, & Pareja, 2016; Rasmann et al., 2005). However, multiple members of the phytobiome contribute to the fluctuation, be it disruption or amplification, of a trophic cascade in a natural environment (Dicke, 2016). The experiments presented here show how a subtropical tree's response to infection with a bacterial pathogen, *C*Las, affects interactions in the phytobiome extending to the third trophic level, eliciting what was previously thought to be solely an HIPV response to root damage in citrus (Ali et al., 2011).

Both root‐feeding herbivores and EPNs were more attracted to *C*Las‐infected roots than to healthy plants in this investigation (Figures 1 and 4). Also, nonfeeding *D. abbreviatus* larvae were more likely to be infected with EPNs when occurring adjacent to *C*Las‐infected tree roots than roots from uninfected trees (Figure 6). Based on our results, this attraction appears to be linked to the inducible release of pregeijerene from infected plant roots (Table 1). This cue has been previously shown to mediate EPN host location in this system, affecting the behavior of a broad spectrum of EPN species (Ali et al., 2010, 2011). The results presented in this study indicate that this HIPV is also released upon plant stress unrelated to insect feeding. Here, we discuss the implications of this finding with regard to belowground tritrophic interactions and the possible causes of “redundant” plant response to distinct stressors.

Information transmitted via chemicals in the soil depends on the ability of the receiver to select relevant information among the noise (Jablonka, 2002). EPNs use many cues to forage for their hosts in the soil environment; cue is used here because of mounting evidence of the adaptability of EPNs to quickly learn from infochemicals (Rasmann et al., 2012; Willett et al., 2015). The most reliable of these host‐location cues is emission specific and related to the feeding or absolute presence of potential host insects (Turlings, Hiltpold, & Rasmann, 2012). HIPVs are released in response to insect feeding and are used by EPNs as host‐location cues in the soil; this has been shown in multiple systems.

Our data further implicate pregeijerene in directional foraging toward *C*Las‐infected plants. Previous research demonstrated that pregeijerene attracts multiple species of native EPNs at ecologically relevant doses in the laboratory and within at least two distinct agroecosystems (Ali et al., 2012). Citrus has a complicated breeding history (Liu, Heying, & Tanumihardjo, 2012) and has been considered a cultivated plant for many years making postulation about how this might impact naturally occurring trees beyond the scope of this study. However, in ancestral hybrid rootstocks, pregeijerene is released constitutively rather than as an induced response observed in cultivated rootstocks (Ali et al., 2011). Furthermore, in nature citrus does not occur in monoculture and disease incidence is likely infrequent (Liu et al., 2012). Due to rapid spread of HLB, nearly 100% of trees may become infected in citrus groves where *C*Las is present. Therefore, our current view of how HIPVs may shape EPN communities in cultivated citrus is influenced by these new findings. In monocultures where HLB disease incidence is prevalent, pregeijerene, a chemical we show not to be solely induced by herbivory, could potentially attract EPNs to trees lacking a suitable host to infect. This would represent a dead end that does support EPN reproduction.

It is possible that redundant release of this previously considered specific EPN host‐seeking cue could have negative consequences for successful EPN reproduction in areas where HLB disease occurs. Alternatively, given that the herbivores were also attracted to infected plants, the redundancy of this cue may be counterbalanced in citrus groves with high *C*Las infection through increased dependence on pregeijerene‐alternative cues relating to an insect's location such as carbon dioxide or scents from insect frass. Furthermore, it is possible that nematodes responded to other signature cues released by infected trees, in addition to pregeijerene, which were undetected here. Also, differential attraction to this cue based on relative ratios of pregeijerene to its degraded form, geijerene (Ali et al., 2010), may indicate presence of the herbivore over a time course following initiation of herbivory, which is a hypothesis that warrants further testing.

Consistent with aboveground studies demonstrating vector attraction to infected plants (Mann et al., 2012; Mauck, De Moraes, & Mescher, 2010) and investigations with *Arabidopsis* indicating benefit to the herbivore from bacterial infection of the plant (Cui et al., 2002; Groen et al., 2013), the root herbivore species investigated here were also more attracted to infected plants than to uninfected plants. This response appeared to be mediated by pregeijerene (Table 1), which was attractive alone to larval weevils, when presented at an optimal dosage (Figure 7). We postulate that the root herbivores are attracted to pathogen‐infested plants because such plants are weakened by bacterial infection. The *C*Las pathogen is phloem‐limited (da Graca et al., 2016) and is known to specifically accumulate in the roots following inoculation into the plant by the psyllid vector (Johnson, Wu, Bright, & Graham, 2013). It is also possible that there is a nutritional benefit to herbivores that feed on HLB‐infected plants given accumulation of callose in the phloem of diseased trees (Kim, Sagaram, Burns, Li, & Wang, 2009) and/or weakened defense response (Nwugo, Duan, & Lin, 2013). This may impact the phytobiome by resulting in development of higher quality insect hosts for EPNs to generate greater numbers of progeny, as occurs with infected cadavers (Barbercheck, Wang, & Hirsh, 1995; Hazir et al., 2016). It may eventually become detrimental to EPN populations due to a coinciding increased incidence of nematophagous species (Duncan et al., 2007) if these members of the community become more associated with pathogen‐infected than uninfected plants (Figure 6). This hypothesis deserves future investigation.

Why would the plant coincidentally release the same volatile organic compound in response to a bacterial pathogen as occurs due to herbivore feeding? It is possible the compound serves other purposes in the root system not explored here, such as antimicrobial activity (e.g., Huang et al., 2012). The lack of specific recognition of *C*Las infection versus herbivore feeding by the plant may be caused by citrus cultivation practices, or pregeijerene release may be a more general stress response by citrus than previously thought. The use of pregeijerene as an HIPV host‐seeking cue by EPNs may have evolved following cultivation of citrus in monoculture 360 years ago (Liu et al., 2012). The inducible release of pregeijerene following herbivore damage in the commercial hybrid rootstock investigated here and constitutive release from the hybrid's parent (Ali et al., 2011) support this hypothesis. Furthermore, commercially cultivated citrus hybrid scions are grafted onto hybridized rootstocks that are compatible, but have largely unresolved phylogeny (Garcia‐Lor et al., 2013). Thus, there is an unknown degree of previously evolved defense trait compatibility between scions and rootstocks. While the scion and rootstock form a fully functional plant, there could be molecular incompatibilities affecting the plant's ability to recognize and differentiate intraplant signals (Aritua, Achor, Gmitter, Albrigo, & Wang, 2013); this may produce two varying sets of plant‐derived molecules in response to infection in the upper portion of the plant versus herbivore damage to the rootstock (Goldschmidt, 2014; Heil et al., 2012). There is evidence, however, that shoot defenses appear to vary in a rootstock‐dependent manner (Agut, Gamir, Jaques, & Flors, 2016). Further investigation is warranted to determine how various scion and rootstock combinations may differentially respond to herbivore damage versus bacterial pathogen infection.

Plants express traits that can attract indirect defenses, such as EPNs, which in turn affects other members of their community. The result of these cascading interactions may benefit the plant so as to limit damage from herbivory (Mumm & Dicke, 2010a). It is reasonable to assume that selection pressure should favor these traits and they may evolve in agroecosystems much more rapidly than in nature as plants are forced into monoculture; redundancy of trait expression throughout an area may allow for quicker attuning to the defense cry by third‐trophic‐level predators and parasites (Dicke & Baldwin, 2010).

In the case of forcing perennially flushing citrus trees into monoculture, it is unclear how the plant's response to infection with the *C*Las pathogen may benefit or disadvantage other members of the phytobiome. The HLB pathosystem is a relatively “new” disease, and it is still unclear whether the pathogen occurred innocuously among vector populations before negatively affecting cultivated monocultures of citrus (da Graca et al., 2016). Constitutive release of pregeijerene from ancestors of cultivated citrus, we speculate, may have evolved as an HIPV to “co‐opt” EPNs as an indirect defense against herbivory (Ali et al., 2011). However, our current results show how infection of citrus with a phloem‐limited bacterial pathogen causes coincident induced release of this HIPV from a commercially cultivated hybrid citrus rootstock. Thus, the benefit of emitting this HIPV to the plant with regard to a belowground tritrophic interaction involving a root herbivore and the EPNs that prey upon it is currently unclear. Looking more broadly into the phytobiome, we found evidence that bacterial infection actually attracts the root herbivores themselves with this HIPV. It is therefore unclear whether the plant utilizes this as a specific cue for defense against herbivore feeding or whether it is an incidental cue released without the “signaling” specificity that was previously postulated (Ali et al., 2010).

In conclusion, it is becoming clear that at least in the citrus model system, EPNs respond to many induced plant cues (Filgueiras et al., 2016). Their adaptability may be due to behavioral plasticity guided by both intra‐ and interspecific communication and short‐ and long‐term memory (Willett et al., 2015). EPNs are therefore able to tailor their behavioral responses to dynamic changes in the subterranean environment and respond to ephemeral cues associated with their hosts rather than relying upon “signature” cues previously thought to be consistently associated with their hosts. Future research should continue to investigate the effect of stressor complexes to seek patterns within these multilayered interactions.

## CONFLICT OF INTEREST

None declared.

## AUTHOR CONTRIBUTIONS

LLS and KSP‐S collected the data. KSP‐S provided materials. MJR and LLS wrote the manuscript. MJR and XM analyzed the data. All authors contributed to study design and interpretation.
